# Healthy eating among people with type 2 diabetes in the Brazilian Amazon: a qualitative study in primary health care

**DOI:** 10.1590/0102-311XEN175125

**Published:** 2026-08-21

**Authors:** Tiago Assunção dos Santos Farias, Thalyta Mariany Rêgo Lopes Ueno, Wagner Ferreira Monteiro, Hércules Lázaro Morais Campos, Elisa Brosina de Leon

**Affiliations:** 1 Faculdade de Educação Física e Fisioterapia, Universidade Federal do Amazonas, Manaus, Brasil.; 2 Faculdade de Ciências da Saúde, Universidade do Estado do Amazonas, Manaus, Brasil.; 3 Instituto de Saúde e Biotecnologia, Universidade Federal do Amazonas, Manaus, Brasil.; 4 Programa de Pós-graduação Interdisciplinar em Estudos Rurais, Universidade Federal dos Vales do Jequitinhonha e Mucuri, Diamantina, Brasil.

**Keywords:** Primary Health Care, Qualitative Research, Vulnerable Populations, Healthy Lifestyle, Noncommunicable Diseases, Atenção Primária à Saúde, Pesquisa Qualitativa, Populações Vulneráveis, Estilo de Vida Saudável, Doenças Não Transmissíveis, Atención Primaria de Salud, Investigación Cualitativa, Poblaciones Vulnerables, Estilo de Vida Saludable, Enfermedades No Transmisibles

## Abstract

This study aimed to explore perceived barriers and facilitators to healthy eating among individuals with type 2 diabetes mellitus and community health workers (CHWs) in primary health care in the Brazilian Amazon. This qualitative study used a participatory approach. Data were collected by World Café discussion groups and semi-structured interviews with type 2 diabetes mellitus patients and CHWs. Thematic network analysis was used to find intrapersonal, interpersonal, and environmental factors influencing dietary behaviors. This study included 64 participants - 47 type 2 diabetes mellitus patients and 17 CHWs in the Brazilian Amazon. Key barriers to healthy eating included financial constraints, lack of family support, and limited access to qualified health professionals such as nutritionists. Additional environmental challenges included seasonal food shortages and poor access to markets. Facilitators included personal motivation, dietary awareness, family support, and local food cultivation. Although the Amazon has a rich biodiversity conducive to healthy eating, socioeconomic and environmental conditions often prevent people with diabetes from accessing healthy food. The findings underscore the need for culturally appropriate, community-based strategies involving multidisciplinary teams to improve dietary adherence in low-resource settings.

## Resumo

Este estudo explorou as barreiras percebidas e facilitadores para uma alimentação saudável entre indivíduos com diabetes mellitus tipo 2 e agentes comunitários de saúde (ACS) em um ambiente de atenção primária na Amazônia brasileira. Este estudo qualitativo utilizou uma abordagem participativa. Dados foram coletados por meio de grupos de discussão do *World Café* e entrevistas semiestruturadas com pacientes com diabetes mellitus tipo 2 e ACS. Análise de Redes Temáticas foi aplicada para identificar fatores intrapessoais, interpessoais e ambientais que influenciam os comportamentos alimentares. O estudo incluiu 64 participantes: 47 pacientes com diabetes mellitus tipo 2 e 17 ACS na Amazônia brasileira. As principais barreiras para uma alimentação saudável incluíram restrições financeiras, falta de apoio familiar e acesso limitado a profissionais de saúde qualificados, como nutricionistas. Desafios ambientais adicionais incluíram escassez sazonal de alimentos e acesso precário aos mercados. Facilitadores incluíram motivação pessoal, conscientização alimentar, apoio familiar e cultivo local de alimentos. Embora a região amazônica possua uma rica biodiversidade que favorece uma alimentação saudável, condições socioeconômicas e ambientais frequentemente impedem pessoas com diabetes de acessar alimentos saudáveis. Os achados ressaltam a necessidade de estratégias culturalmente adequadas e baseadas na comunidade, envolvendo equipes multidisciplinares, para melhorar a adesão alimentar em ambientes de poucos recursos.

## Resumen

Este estudio exploró las barreras percibidas y los facilitadores para una alimentación saludable entre personas con diabetes mellitus tipo 2 y agentes comunitarios de salud (ACS) en un entorno de atención primaria en la Amazonia brasileña. Este estudio cualitativo utilizó un enfoque participativo. Se recopilaron los datos a través de grupos de discusión del *World Café* y entrevistas semiestructuradas con pacientes con diabetes mellitus tipo 2 y ACS. Se aplicó un Análisis de Redes Temáticas para identificar los factores intrapersonales, interpersonales y ambientales que influyen en los hábitos alimenticios. El estudio incluyó a 64 participantes: 47 pacientes con diabetes mellitus tipo 2 y 17 ACS en la Amazonia brasileña. Las principales barreras para una alimentación saludable incluyeron restricciones financieras, falta de apoyo familiar y acceso limitado a profesionales de la salud calificados, como nutricionistas. Otros desafíos ambientales observados incluyeron la escasez estacional de alimentos y el precario acceso a los mercados. Los facilitadores incluyeron la motivación personal, la conciencia alimentaria, el apoyo familiar y el cultivo local de alimentos. Aunque la región amazónica tiene una rica biodiversidad que promueve una alimentación saludable, las condiciones socioeconómicas y ambientales suelen impedir el acceso de personas con diabetes a alimentos saludables. Los hallazgos resaltan la necesidad de estrategias culturalmente adaptadas y basadas en la comunidad, que involucren equipos multidisciplinarios, para mejorar la adherencia alimentaria en entornos con recursos limitados.

## Introduction

The Brazilian Amazon (rich in natural resources, particularly in its diverse fish and fruits) could support excellent health and nutrition by the adequate intake of essential nutrients ^1^ ^,^ ^2^. However, prevailing social, economic, and cultural conditions and documented health and nutrition deficiencies undermine this potential in the region ^3^.

The Amazon, despite its unique biodiversity, is undergoing a complex nutritional transition driven by rapid, disordered urbanization and increased consumption of ultra-processed foods. Regional singularities shape this reality since geographic isolation and dependence on water cycles determine access to food. Extreme climatic events (such as the recently documented severe droughts and large floods) have isolated riverine and rural communities, compromising supply logistics and increasing the cost of fresh food ^4^ ^,^ ^5^ ^,^ ^6^. This pattern is especially pronounced in economically vulnerable urban areas, in which financial constraints often override food quality considerations at the point of purchase ^5^ ^,^ ^6^.

The growing prevalence of chronic conditions, particularly type 2 diabetes mellitus, has heightened the vulnerability of the population, posing complex challenges for patients, families, and health professionals ^7^ ^,^ ^8^. As a result, type 2 diabetes mellitus has become a priority in the Brazilian primary health care (PHC) system ^9^, which plays a key role in preventing acute and chronic complications ^10^ ^,^ ^11^.

In type 2 diabetes mellitus, nutritional and dietary interventions are central to improving care strategies, emphasizing the importance of healthy eating throughout the life course ^9^ ^,^ ^11^ ^,^ ^12^. However, adopting healthy eating habits remains difficult. Non-adherence to dietary and treatment guidelines can compromise disease management, weaken professional-patient relationships, and raise healthcare costs ^13^. Effective behavioral change requires patient motivation to address barriers and leverage facilitators ^14^. Long-standing beliefs, including skepticism toward conventional health systems, may limit the use of available services. For better disease management and community engagement, health interventions must integrate and respect local knowledge and cultural practices ^15^ ^,^ ^16^ ^,^ ^17^.

Although the scientific evidence consistently supports the protective role of a balanced, fiber-rich, low-fat diet combined with regular physical activity in reducing the risk of type 2 diabetes mellitus ^18^ ^,^ ^19^ ^,^ ^20^ ^,^ ^21^, no increase in the acquisition of healthy behaviors (including dietary ones) ^18^ occurs despite the participation of patients in activities to manage type 2 diabetes mellitus in primary care.

This region shows cultural diversity, economic challenges, unique food traditions, and logistical issues that may impact the availability and consumption of healthy foods. Thus, this study aimed to explore perceived barriers and facilitators to healthy eating in individuals with type 2 diabetes mellitus and primary health care community health workers (CHWs) in the Brazilian Amazon.

## Materials and methods

### Type of study

A qualitative approach with a participatory interface ^22^ was used in this study, embedded in a project titled *Intervention Led by Community Health Workers for the Management of Type 2 Diabetes in the Interior of Amazonas*. The consolidated criteria for reporting qualitative research checklist was used in this research.

### Study location and population

This study was conducted in Iranduba, a municipality located 38.1km from Manaus, in inner Amazonas State, Northern Brazil. The municipality has six PHC units throughout its urban area, all within a 30km radius of the municipal health department headquarters ^23^.

The municipality of Iranduba was selected for representing the cultural and sociodemographic characteristics of Brazilian Amazonian municipalities ^24^. The sample comprised 64 participants (47 patients with type 2 diabetes mellitus and 17 CHWs), predominantly residents of the municipal seat but with strong ties to rural areas and surrounding communities.

The study population included individuals with type type 2 diabetes mellitus and CHWs. Active CHWs affiliated with the six primary health care units within the municipality urban perimeter (excluding those on leave or vacation) were included. Adult (≥ 18 years) patients with type 2 diabetes mellitus who had been diagnosed at least six months prior to this research, were linked to the same PHC units, and followed by participating CHWs were included in this research. Those with physical, intellectual, or communicative limitations that prevented participation were excluded. The same criteria were applied for the individual interviews in addition to the requirement of prior non-participation in the World Café (to broaden the diversity of experiences; https://theworldcafe.com/). These criteria ensure methodological coherence and enable access to diverse experiences relevant to the studied phenomenon.

### Data collection

Data were collected using the World Café technique and semi-structured interviews. World Café sessions were held separately for patients with type 2 diabetes mellitus (in the morning) and CHWs (in the afternoon) on different days, with day 1 for barriers and day 2 for facilitators, with each group participating in two meetings collectively titled “*Prosa e Café*”. The World Café sessions were held at a location outside the PHC units, which was strategically selected for its spacious, welcoming, and neutral environment (essential to the method). The first session explored perceived barriers to healthy eating and physical activity, whereas the second addressed facilitators. Participants also completed a sociodemographic questionnaire and concluded their discussions with a plenary session to share ideas ^25^ ^,^ ^26^.

Participants were recruited with the municipal administration. Initially, the research team met with the coordination of the municipal health department, then visiting the six PHC units to present their project to the coordinators and CHWs. Then, an online meeting was we held. A WhatsApp group was created to facilitate communication and solve doubts. The CHWs, knowing their assigned population, compiled a list of 50 patients with type 2 diabetes mellitus and directly invited them during home visits or routine contacts. Only 32 patients and 17 CHWs participated in this research. Reasons for non-participation included personal or professional obligations, health problems, or refusal.

To complement and further the conclusions of the group discussions, semi-structured home interviews were conducted with patients with type 2 diabetes mellitus who participated in the World Café session. The CHWs provided contact information for 25 eligible patients, of whom 15 agreed to participate. The interview script was developed specifically for the individual stage and structured into two parts: the first contained socioeconomic questions to characterize the participants, whereas the second part consisted of open-ended questions that had been designed to deeply explore participants’ lived experiences, the meanings attributed to care, and the perceived barriers to physical activity. The interviews were recorded on tablets provided by the researchers and securely uploaded to a Google Drive folder daily. The interviewees were identified using the codes U1-U15. Theoretical saturation was adopted as the criterion for concluding data collection, defined as the point at which no new insights emerged to alter the understanding of the studied phenomenon ^27^ ^,^ ^28^ ^,^ ^29^.

### Research team and data analysis

A trained research team facilitated the World Café sessions and conducted the semi-structured interviews. The research team included a postdoctoral researcher, master‘s students, undergraduate physical therapy students, and the study coordinator of the overarching project. They were trained to guide discussions at the “*Prosa e Café*” tables and conduct home interviews with individuals with type 2 diabetes mellitus.

Data from the World Café − recorded on tablets and in video format − were transcribed using Reshape and organized with ATLAS.ti (http://atlasti.com/). A hermeneutic unit was created to support analysis. The interviews were transcribed and reviewed, after which an initial set of codes was deductively developed. The research team then collaboratively refined the coding and conducted a second round of analysis to find patterns and themes. This process built a thematic network on barriers and facilitators to the adoption of healthy eating, comprising 13 basic codes for barriers and 15 for facilitators.

To visually represent the data within the thematic network, the thickness of the lines connecting codes indicates their frequency across participant narratives. Thicker lines indicate more frequent mentions, highlighting the themes that were most salient in the dataset ^30^. The barriers and facilitators for adopting healthy habits in people with type 2 diabettes mellitus were categorized in light of the concepts by Whittlemore et al. ^31^, which highlight multiple levels of influence that can affect behavior change. The levels of influence include intrapersonal, interpersonal, and environmental factors.

### Ethical considerations

This study complied with ethical guidelines in *Resolutions n. 466/2012* and *n. 510/2016* of the Brazilian National Health Council and the National Research Ethics Commission. It was approved by the Research Ethics Committee of the Federal University of Amazonas (approval number 5.931.419) and received additional authorization from the Municipal Health Department of Iranduba. All participants signed an informed consent form and granted permission for the use of their image and voice. Participants were anonymized using the codes US1-US32 (patients) and A1-A17 (CHWs).

## Results

**Figure 1:**
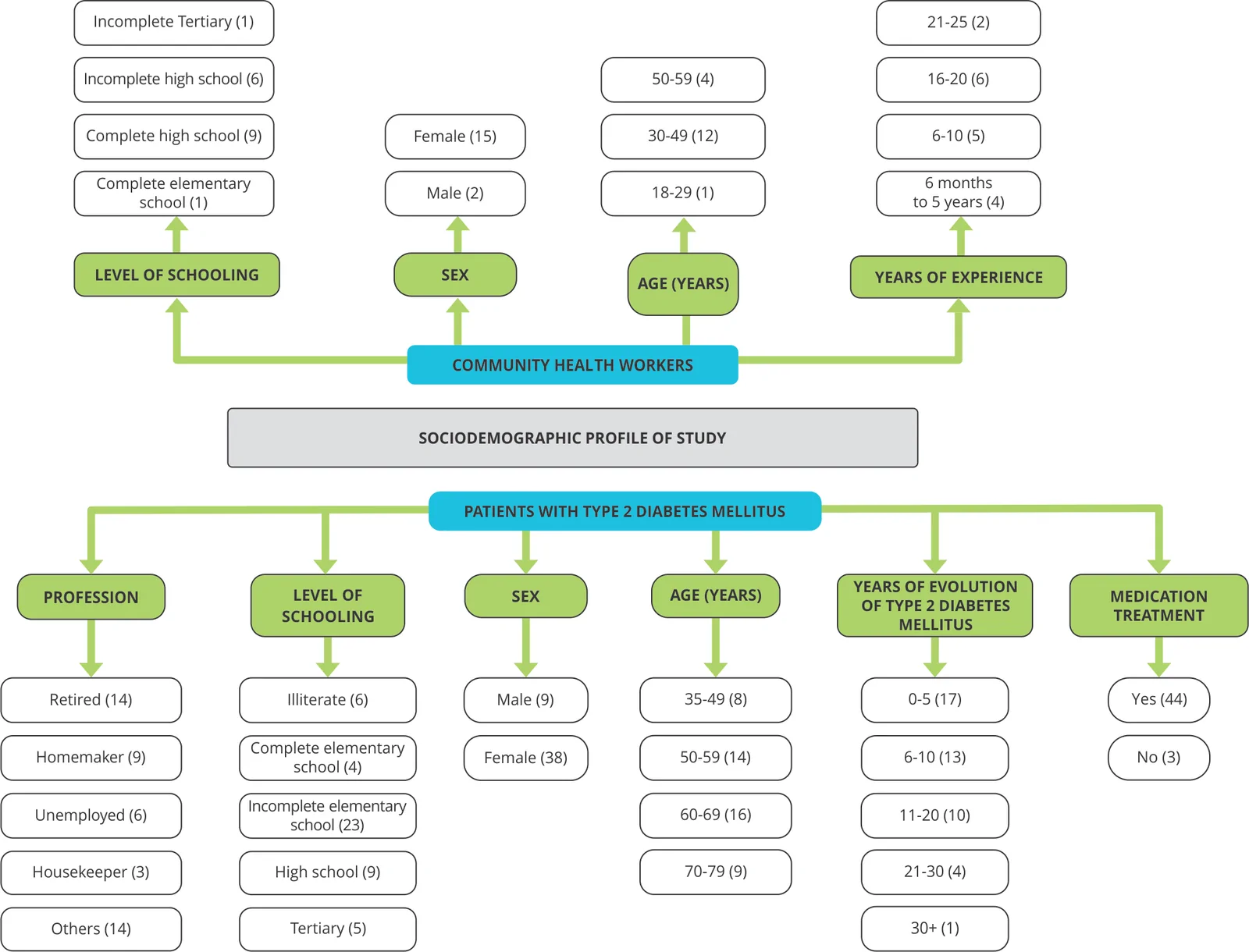
Characterization of the research participants.

This study included 64 participants: 47 with type 2 diabettes mellitus and 17 CHWs. Among them, 32 patients and 17 CHWs participated in the “*Prosa e Café*” dynamics, whereas the remaining 15 type 2 diabetes mellitus patients participated in semi-structured interviews. Most participants were women both among the CHW and patients, totaling 81.25% (52) of the sample. Their ages ranged from 30 to 79 years. The most common level of education among patients was incomplete elementary school 71.88% (23), whereas among CHWs, complete high school predominated: 52.94% (9) (Figure 1).

**Figure 2:**
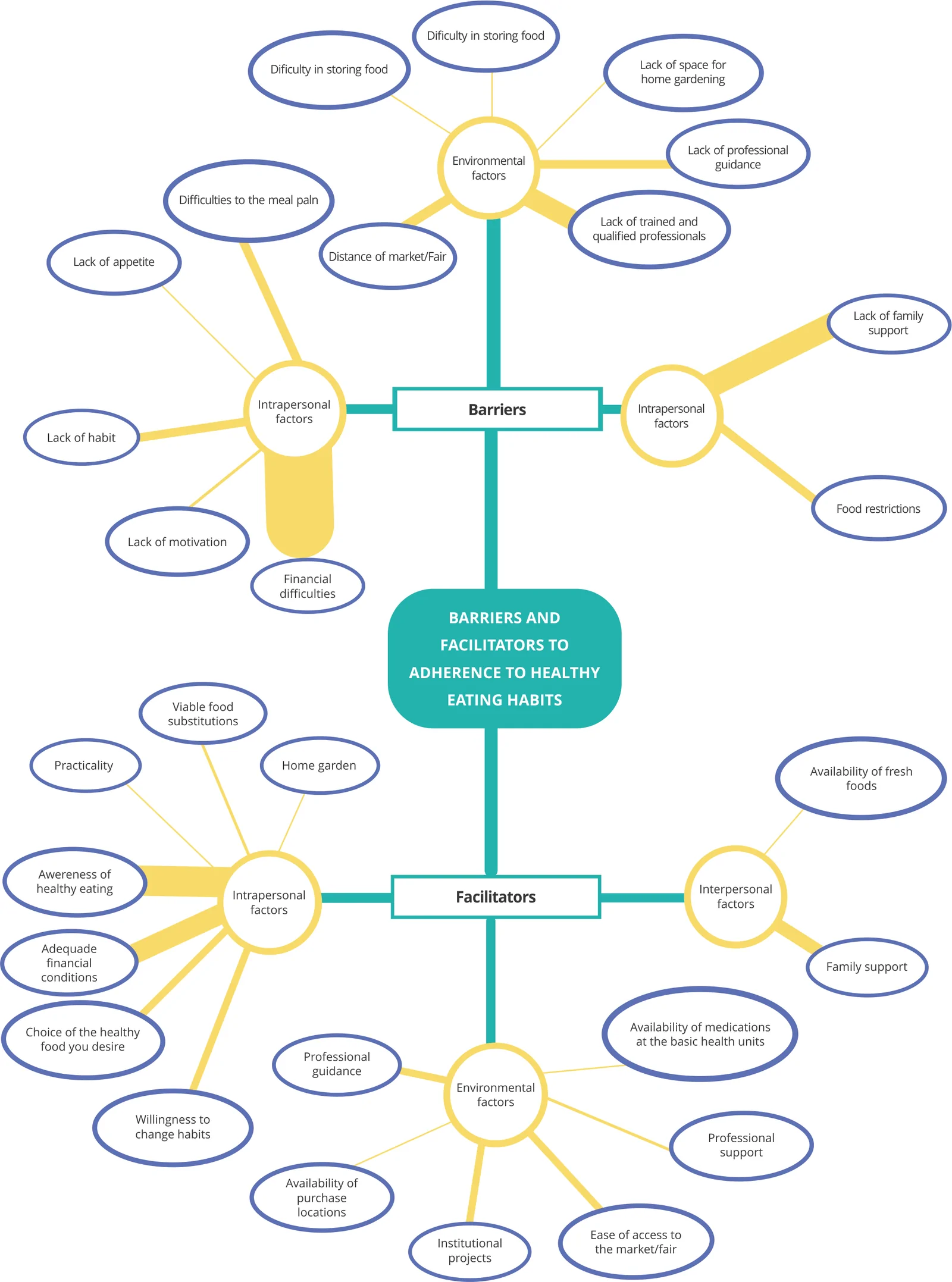
Structuring the thematic network of “*Prosa e Café*”.

The results of this study followed the 13 and 15 basic themes for barriers and facilitators, respectively. This research identified and organized them into three themes: “Intrapersonal factors”, “Interpersonal factors”, and “Environmental factors”. The grouping of the organizational themes culminated in the overarching theme “*Barriers and Facilitators to Adopting Healthy Eating Habits*”, the thematic network of which synthesizes the difficulties and facilitators type 2 diabettes mellitus patients experience in adopting healthy eating habits based on the perspectives of patients and professionals living in a municipality in inner Amazonas (Figure 2).

### Barriers to adherence to healthy eating habits

The first organizational theme, titled “Intrapersonal factors”, is composed of five basic themes that address the internal difficulties type 2 diabettes mellitus patients face when trying to adhere to a healthy diet. These challenges arise due to a lack of financial resources, inappropriate food cravings, unfamiliarity with certain foods or eating practices, and a lack of motivation.

“*Many times, their money is only for household expenses; there’s nothing left to buy food. Everything is also very expensive. The price of meat per kilo is outrageous, and at home, we prefer sausage instead*”(A4).

“*My difficulty is having to choose at the market between one vegetable and one fruit or having to pay for a consultation or medication*” (US5).

“*It is difficult to eat healthily because we are used to eating everything. Additionally, during the drought, an orange costs three reais, whereas Tang costs one real and is much more practical*...” (US2).

Composed of two basic themes, the second organizational theme, titled “Interpersonal factors”, addresses how interpersonal relationships affect non-adherence to healthy eating habits. These behaviors are related to the lack of family support and dietary restrictions.

“*If there is a diabetic in my house* [whereas the others are not], *do you think they will choose to eat what they shouldn’t? They will eat what everyone else is eating there*” (A2).

“*It was also reported that sometimes we cook at home, making tasty food for our child and husband while we have to eat that bland food. Grilled fish, just with salad, without a pleasant flavor. Many people mentioned this*” (US3).

The third organizational theme refers to environmental factors. It consists of six basic themes. Patients reported challenges in their communities due to environmental conditions and the management of health and social services, which impact adherence to healthy eating habits. This included issues such as poor sanitation, long distances to purchasing locations, lack of trained professionals, among others.

“*Distances prevent them from having a more adequate diet. When they leave the PHC, the physician gives them guidance on seeking help, but they don’t go because of the distance, and many times they don’t want to take money out of their pockets to pay for a motorcycle taxi*” (A10).

“*There is no nutritionist available for consultations on proper nutrition*” (U12).

“*This drought is severe; fish is expensive and hard to find and so are fruits. The smoke from the fires complicates everything. Good food is becoming very expensive; so we end up buying what we can afford*...” (U11).

### Facilitators for adherence to healthy eating habits

The first organizational theme, titled “Intrapersonal factors”, is composed of seven basic themes that refer to how individual characteristics influence a person’s adherence to healthy eating practices. This includes personal attitudes and perceptions about healthy eating, financial conditions, food preferences, and oscitations, as well as the possibility of planting and cultivating one’s own food.

“*I used to eat everything whole grain, and my diabetes wouldn’t go down. I started eating only natural foods like vegetables and fruits from the backyard and I managed to lower it*” (US7).

“*It makes it easier to have a basic diet because everyone knows that fruits are expensive. Here in Iranduba, they are available, but only if you have a good income to buy them*” (A1).

Composed of two basic themes, the second organizational theme, called “Interpersonal factors” addresses how external interactions contribute to the goal of healthy eating. These behaviors are related to family support and the availability of resources for maintaining a healthy diet.

“*So, here we have an abundance of fruits, vegetables, and fish. When you can’t buy fish, you go fishing or your nephew goes and fishes. When you can’t afford to buy a fruit, you have it in your backyard*” (US3).

“*When I don’t have money, I can’t make purchases; sometimes my daughters buy and give me some. My son, when he’s here, doesn’t forget about me* (...) *my food is never lacking! He helps as much as he can. My sons-in-law also make purchases, and that’s how it is*” (U9).

The third organizational theme, “Environmental factors”, consists of six basic themes on how the environment and external conditions affect the ease of eating better and healthier. Participants reported specialized professional follow-up, the presence of institutional projects, ease of access to purchasing locations, among others.

“*Patients have consultations; they receive care at the PHC. So, if this action included all professionals* (...) *not just nurses, CHWs, and technicians but also a nutritionist and a physical educator providing guidance, I am sure that they would indeed change their eating habits*” (A1).

“*It would facilitate actions within Hiperdia* [Registration and Monitoring Program for Patients with Hypertension and Diabetes] *with nutritional follow-up*” (A9).

“*It would facilitate having a fruit and vegetable market in the community, for example, with affordable prices*” (U6).

## Discussion

This study explored perceived and experienced barriers and facilitators to adherence to healthy eating habits among type 2 diabetes mellitus patients and PHC CHWs. Participants’ significant statements evinced factors that directly and indirectly influence adherence to this healthy habit.

The high prevalence of low education in the sample (71.88%) suggests low health literacy, which may impose substantial barriers to interpreting nutritional guidelines and self-managing type 2 diabetes mellitus ^7^. This limitation makes patients dependent on simpler communication from PHC units ^13^. However, high staff turnover and shortage of specialists weaken the healthcare system of small municipalities. The lack of nutritionists, previously reported by the research team ^18^, disrupts the continuity of care and invariably overburdens CHWs, who mediate dietary guidelines without adequate technical support, compromising the accuracy of the information ^14^ ^,^ ^32^.

In addition to this scenario, the cultural competence of professionals becomes indispensable. This factor reinforces the need for guidelines adapted to regional specificities, such as the frequent consumption of cassava flour and Amazonian fish, lest the recommendations may become unfeasible given the sociocultural reality of the population ^3^ ^,^ ^33^.

The factors “financial conditions” and “family support” appeared as facilitators and as barriers to adherence to healthy eating habits. Financial difficulty configured the most frequently mentioned barrier in participants’ accounts. This discussion also emerged in Santos et al. ^34^ as the authors emphasized that product prices involve more than individual or family budgeting; they follow economic and political choices that often fail to prioritize healthy eating. Furthermore, economic restrictions on food purchases lead to diets with low consumption of fruits and vegetables and high energy density ^35^. Despite the diversity of fruits and the potential for healthy eating in the Amazon, climatic factors such as droughts and floods affect fresh products, increasing the prices of such foods more than those of ultra-processed ones, which stand out for their convenience ^36^.

Beyond financial barriers, psychological barriers arise that hinder adherence to a healthy diet. This study found a psychological resistance that manifest itself by an aversion to foods considered healthy − which are frequently associated with restrictive and unappealing diets in contrast to a preference for ultra-processed foods, which are often considered more palatable ^37^ ^,^ ^38^. This aversion become stronger within the Amazonian family environment, in which it is traditional to eat meals together. For patients with type 2 diabetes mellitus, adopting a diet unlike that of others generates emotional distress and a feeling of social exclusion ^39^ ^,^ ^40^. It is worth considering that the desire to maintain family cohesion and local traditions − such as the excessive consumption of carbohydrates, especially cassava flour ^3^ ^,^ ^41^ ^,^ ^42^ ^,^ ^43^ − suppresses individuals’ willingness to change their habits.

Droughts, wildfire smoke, and difficulty accessing healthy foods due to scarcity and rising prices further exacerbate the local food situation ^44^. Moreover, the lack of specialized professionals (such as nutritionists) and the absence of culturally adapted guidance contribute to low adherence to healthy eating habits ^11^ ^,^ ^45^. Nutritionists play a crucial role in PHC multidisciplinary teams and in direct contact with the population, promoting care and health actions ^16^. Clinical practice in Amazonian PHC should shift its focus from isolated dietary prescriptions to nutritional counseling that involves the family unit and prioritizes local biodiversity to facilitate adherence ^3^ ^,^ ^4^.

Regarding facilitating aspects, growing one’s own food, specifically in a “home garden”, is of significant importance. The Brazilian Ministry of Health ^46^ states that cultivating food at home represents a substantial stimulus for adequate and healthy eating. Additionally, Silva et al. ^47^ and Dode et al. ^48^ highlight that cultivating food in home gardens supports a healthier, more sustainable diet, reduces the costs of purchasing vegetables, and encourages a more mindful approach to food.

Changing eating habits is a complex process, and overcoming perceived barriers, increasing general awareness about health, strengthening social influence, and self-efficacy may be essential to implementing and sustaining these modifications ^45^. Regarding barriers, factors such as lack of motivation, deficits in self-control or nutritional knowledge, and financial difficulties ^7^ often hinder adherence to healthy eating habits. Concurrently, “awareness of healthy eating” and “willingness to change habits” emerged in this research as intrapersonal facilitating factors. A previous study showed that the Amazonian population with type 2 diabetes mellitus shows low motivation for behavioral change, which impacts disease management ^49^. Autonomous motivation factor is important for regular physical activity and healthy eating practices ^50^.

Regarding interpersonal facilitator aspects, the findings of this study corroborate those of Espinosa et al. ^37^, who found that personal purpose and family support constituted the main facilitators of adherence to healthy eating. Mendes et al. ^20^ and Polhuis et al. ^8^ also addressed family participation, showing that family involvement contributes to adherence and self-management of type 2 diabetes mellitus, especially outside supervised settings.

In addition to the aforementioned factors, the participants in this study also emphasized that professional follow-up and specialized guidance facilitate adherence to a healthy diet. This was also emerged in the statements of participants in Siopis et al. ^51^, who reported that facilitating adherence to healthy eating habits requires improving food and nutritional literacy, providing social and family support, and performing follow-up by nutritionists.

### Limitations

One of the main limitations of this study is its geographic focus as it was conducted in a single municipality in the state of Amazonas, Brazil, which may have compromised the applicability of its findings to other locations. However, previous research in the region suggests that various municipalities in Amazonas share similar demographic and social characteristics ^24^ ^,^ ^52^. Additionally, adopting a qualitative approach in a low-income population offers significant challenges. Participants may feel distrust or hesitate to disclose personal information, especially if they are unsure how their data will be used or if they have had negative experiences with institutions. Furthermore, low literacy and communication difficulties may hinder individuals’ understanding and participation in interviews or focus groups, particularly when discussing complex or unfamiliar topics.

## Conclusion

This research showed that, despite the rich food biodiversity in the Amazon, the management of type 2 diabetes mellitus faces severe structural and environmental barriers. The findings in this study showed that low health literacy (associated with low levels of education) makes patients dependent on technical support the PHC still struggles to fully provide. The lack of specialized professionals, such as nutritionists, weakens direct care and overloads CHWs, who then act as health educators without the necessary multidisciplinary support.

Concomitantly, the local climatic and logistical conditions render access to healthy food as unstable and financially prohibitive for the most vulnerable. Therefore, interventions to manage type 2 diabetes mellitus in the Amazon should avoid focusing solely on generic clinical guidelines. Public policies must retain professionals with cultural competence, value local production (such as home gardens), and consider geographical particularities as central determinants. Overcoming the individualistic view of habit change and adopting an approach that articulates food security, accessible health education, and the strengthening of PHC unit teams is fundamental to reducing health inequities and improving the prognosis of patients with type 2 diabetes mellitus in the Amazon.

## Data Availability

The research data are available upon request to the corresponding author.
